# Armed stem to stinger: a review of the ecological roles of scorpion weapons

**DOI:** 10.1590/1678-9199-JVATITD-2021-0002

**Published:** 2021-09-03

**Authors:** Yuri Simone, Arie van der Meijden

**Affiliations:** 1 CIBIO Research Centre in Biodiversity and Genetic Resources, InBIO, Porto, Portugal.

**Keywords:** Chelae, Scorpions, Venom delivery system, Scorpion weapons

## Abstract

Scorpions possess two systems of weapons: the pincers (chelae) and the stinger (telson). These are placed on anatomically and developmentally well separated parts of the body, that is, the oral appendages and at the end of the body axis. The otherwise conserved body plan of scorpions varies most in the shape and relative dimensions of these two weapon systems, both across species and in some cases between the sexes. We review the literature on the ecological function of these two weapon systems in each of three contexts of usage: (i) predation, (ii) defense and (iii) sexual contests. In the latter context, we will also discuss their usage in mating. We first provide a comparative background for each of these contexts of usage by giving examples of other weapon systems from across the animal kingdom. Then, we discuss the pertinent aspects of the anatomy of the weapon systems, particularly those aspects relevant to their functioning in their ecological roles. The literature on the functioning and ecological role of both the chelae and the telson is discussed in detail, again organized by context of usage. Particular emphasis is given on the differences in morphology or usage between species or higher taxonomic groups, or between genders, as such cases are most insightful to understand the roles of each of the two distinct weapon systems of the scorpions and their evolutionary interactions. We aimed to synthesize the literature while minimizing conjecture, but also to point out gaps in the literature and potential future research opportunities.

## Background

Environments where resources are limited increase the competition among their inhabitants. Gaining an advantage in access to resources over competitors raises an individual’s chances to survive and transmit its genes to the next generation. In animals, conflicts may involve a physical antagonistic struggle between individuals. The features that most define the outcome of such competitive conflicts are usually considered “weapons” [1]. Although many definitions of animal weapons are limited to intraspecific competitions, particularly intrasexual competitions [2-7], we will here use the broader definition of a weapon as proposed by Lane [8]. In her definition, animal weapons are features that constrain the behavior of another individual either through direct harm or other physical disruption in one or more of three fundamental contexts of usage, namely: (i) predation, (ii) defense and (iii) sexual contests. Animal weapons may thus be classified by their context of usage, but could also be organized by their mode of action, form or evolutionary history [8]. In this review, we will organize the literature on scorpion weapons by context of usage. We will first give examples of each context of usage from across the animal kingdom to provide a comparative background for the discussion of scorpion weapons.

### Weapons for predation: prey capture and handling

Weapons are used in predation to seize the prey and reduce its chance to escape by restraining or incapacitating it. The grip of the restraining structures on the body of the prey may be increased by increasing friction, interlocking, penetration, or a combination thereof. Raptorial appendages are therefore often covered with spine-like structures (e.g. praying mantis [9-11], mantis shrimp [11-13] or several orders of arachnids [14]). Birds that feed on flying insects often have bills with serrated edges [15,16]. Similarly, sharp teeth, powerful mandibles and claws allow a firm grip on prey by penetrating it [11]. Some species instead resort to chemical secretions to reduce the mobility of a prey. Secretions may be sprayed onto the body of the prey and glue it to the substrate (comprehensively reviewed in [17]). Other secretions, such as venoms, are injected and act on the nervous system, paralyzing or killing the victim [18]. 

### Weapons for defense

The second main context of usage for animal weapons is defense. Inducing pain or other noxious experiences is one of the most efficient strategies to deter predators from pursuing their intention to assault [19]. To be effective, pain must be caused as quickly as possible, preferably before the predator has inflicted harm on the defending animal. Examples of active counterattacks through mechanical means are bites (most mammals [20], squamates [21], arthropods [22]) or pinches (e.g. crustaceans [23], arachnids including scorpions [24], insects [2,22]), scratching (amphibians [25], mammals [20], squamates [21]), stabbing (e.g. ungulates [2,26], swordfish [27]), urticating bristles (e.g. spiders [28], millipedes [22,29]) and flagellation (e.g. squamates [21,30]). Passive mechanical defenses may include urticating setae (e.g. lepidopteran larvae [28]), spines (e.g. Echinoderms [11,31], mammals [11,32] and fishes [11,33]) or hard and pointed scales (e.g. squamates [34-36]) that can severely harm a predator if it attempts to handle, bite or ingest it [33,34], or at least increase handling time to a point to be unprofitable for the predator [37]. 

Beyond causing mechanical damage, a uniquely rapid and remote way to cause pain is by electric shock (e.g. eels [38], electric rays [39]). Predators can also be deterred by substances that cause pain or are otherwise noxious. Such secretions may be sprayed towards the predator/attacker, like in spitting cobras [40,41], bombardier beetles [1,42,43], vinegarroons [22,44], scorpions [45,46] and some species of millipedes [47] and ants [48]. Alternatively, noxious chemicals may be secreted from glands located on the skin, like in many amphibians [49,50]. Finally, noxious secretions may be delivered into the prey’s body through specialized structures like stingers (e.g. hymenopterans [51], scorpions [52,53] and stingrays [54]), fangs and mouthparts (e.g. centipedes [55], spiders [56] and snakes [57]), spines like in many fishes [11,58,59], nettle cells in cnidarians [60] and bony protrusions in amphibians [61,62]. In these cases, mechanical damage is augmented with a chemical agent.

Other secretions instead have the objective of repelling or confounding rather than hurting a predator. Many insects and arachnids secrete unpalatable quinone or phenolic-based substances [22,43]. Squamates and mustelids can release repelling secretions [63-65], while cephalopods and sea hares confound attackers by spraying ink [66,67].

### Weapons used in sexual contests

Weapons that often develop as secondary sexual characters are used to obtain or defend reproductive resources, and/or to coerce sexual partners. In dyadic fights, morphological weapons are used in stabbing (e.g. many bovids [1,26,68-70], narwhals [71,72], walruses [73], elephants [1], rhinoceros beetles [74]) ramming or pushing (many bovids [1,68-70], dung beetles [1,2]), flipping the opponent over (e.g. stag beetles [1,2,75], tortoises [76]) or grappling (cervids [1,68-70], crabs [77], arachnids [14,78]). The use of chemical weapons to solve intrasexual contests is particularly rare and only known in platypus [79], amphipods [80] and loris [81,82]. 

### The weapons of scorpions

Scorpions belong to one of the eleven extant orders of arachnids and are easily recognizable from the other members of the arachnid class by their special set of weapons. Whereas many animals have a single weapon, scorpions possess two separate weapon systems. The pincers or pedipalps that are oral appendages located at the front of the body, and the venom-carrying stinger or telson at the caudal end of the body. Each of them has a different mode of action: mechanical and chemical, respectively. Both weapons are used in all three contexts of usage: predation, defense and sexual contests. The approximately 2,500 species currently described [83] use these two weapon systems in different ways or to different degrees in each of the three contexts of usage, which is reflected in their morphological diversity.

Scorpions are probably among the most ancient arthropods that made a full transition from water to a land-living lifestyle [84-86]. In addition, their body plan almost did not change since the Silurian (443-419 Mya) [87]. They successfully colonized all the continents except Antarctica, which illustrates their extraordinary capacity to adapt to different and sometimes hostile environments, and the versatility of their body plan.

## Scorpion weapons

### Anatomy and functioning

Knowing the inner structure of an anatomical feature is fundamental to understand its performance and limitations. In this section, we will first review the anatomy literature on the two weapons systems of scorpions: their pincers or pedipalps, and their venom delivery system consisting of the telson at the end of the flexible metasoma. Particular focus will be given to the musculoskeletal system and how it is linked with the structure’s role as a weapon in different contexts. We will only mention other, non-weapon functions, such as the sensory function of both the pedipalps and metasoma, in passing. A separate paragraph will be then dedicated to the production, composition and evolution of scorpion venom. 

### Overall anatomy

**Figure 1.**
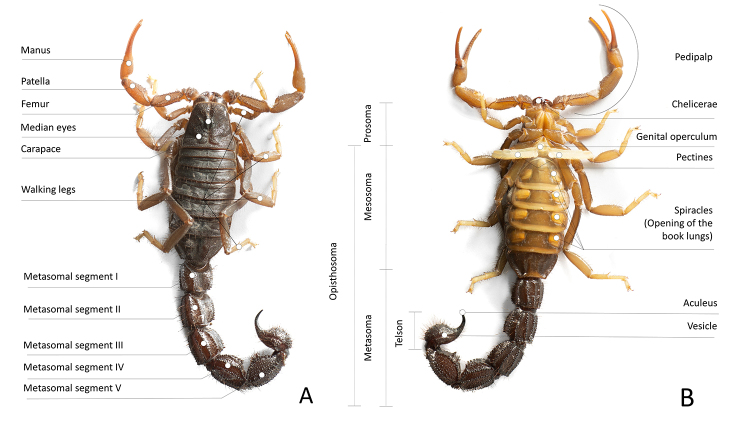
Overall anatomy of a scorpion (*Parabuthus transvaalicus,* Buthidae). **(A)** Dorsal view. **(B)** Ventral view.

As in all arachnids, the scorpion body can be divided in two tagmata: prosoma and opisthosoma (Figure 1). The prosoma works functionally like a head, containing the several sensory organs and major ganglia of the nervous system. All six pairs of appendages are attached to the prosoma. The first pair is used for feeding and forms the mouth parts or chelicerae. The second pair forms the pedipalps ending in the pincers or chelae. The other four appendages are the walking legs, used for locomotion. The body beyond the prosoma, the opisthosoma, is subdivided in mesosoma and metasoma. The mesosoma is the anterior portion of the opisthosoma and contains the sexual organs, the specialized sensorial pectines, four pairs of book lungs, the cardio circulatory system, and the post-prosomal portion of the digestive tract. The metasoma is a tail-like elongation of the body containing the hindgut and carrying the venomous stinger (telson).

### The pincers

The pedipalps are modified appendages developing very early in embryogenesis. Initially situated posterior to the cephalic lobes, pedipalp lobes gradually move anteriorly during the later stages of the embryonic development. After the posterior-anterior migration, the segments forming the pedipalps are recognizable [88,89].

**Figure 2.**
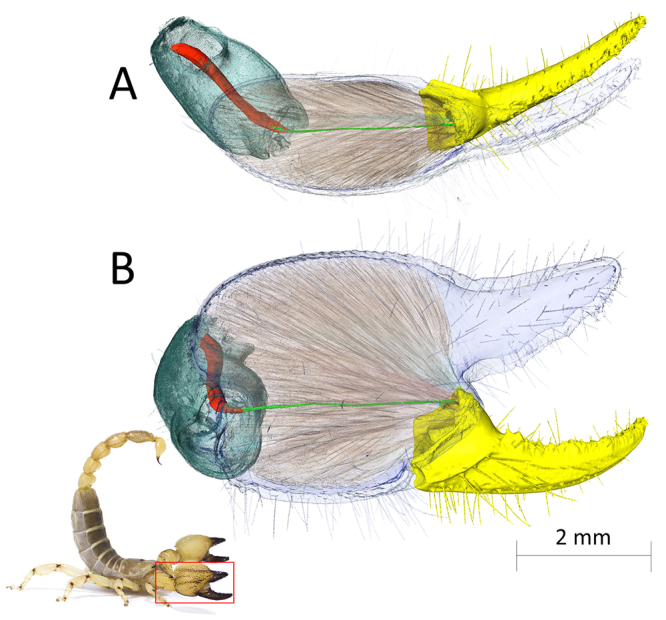
Rendering of the chela closing muscles of the species *Scorpio maurus* (Scorpionidae) from synchrotron scan data. **(A)** Dorsal view. **(B)** Lateral view. Apodemes connecting the closing muscle in the manus to the movable finger are not shown. Only the ligament connecting the closing muscle in the patella to the movable finger is shown. Movable finger (yellow), cuticle of the manus (transparent blue), manus closing muscles (transparent orange), closing muscle in the patella (red), ligament connecting the closing muscle in the patella to the movable finger (light green), cuticle of the patella (dark green).

The last two segments of the pedipalp, namely the manus (or tibia) and the movable finger (or tarsus) form the chela. The manus includes the fixed finger and most of the closing muscles [90-92] (Figure 2). The movable finger acts as a first-class lever system; it rotates on a fixed axis formed by two joints located at the antero-ventral side of the manus, determining the axis of rotation for the opening and closing of the chela. Although scorpion musculature was first described by Lankester [93], pedipalp musculature was not included. Gilai and Parnas [90] reported the existence of three main bundles of closing muscles in the manus of Leiurus quinquestriatus (Buthidae). Dubale et al. [92] recognized eight muscle bundles in the “tarsus depressor” muscle of the manus in a single specimen belonging to the genus Heterometrus (Scorpionidae). Another closing muscle is located in the next proximal segment, the patella [90,92,94]. This muscle is composed of long fibered bundles which are mechanically connected to the movable finger by a long ligament [90]. In the patella, muscles that adduct and abduct the chela in the frontal plane are also present [95].

Contrary to crustaceans [96,97], scorpion chelae do not have opening muscles [90-92], and the movable finger abduction is due to the elastic recoil of resilin in the joint [91,94,98,99], the increasing of hydraulic pressure in the manus and an elastic snap-like recoil given by sclerotized plates (arthrodial sternites) located the dorso-posterior interface of the movable finger and the manus [100].

On the surface of the cuticle of the chela, several hair-like structures with chemo- and mechano-sensorial functions are present: trichobothria, located on the whole surface of both chelae, and a little group of sensilla on the tip of the fixed finger, known as the constellation array. For an excellent description and images of these structures, please refer to [101,102]. Trichobothria are important for environmental sensing and detection of air-borne stimuli [102], while the constellation array seems to play a role in the detection of chemical cues [101,103]. Furthermore, trichobothria placement patterns are extensively used as important taxonomic traits [104-106]. The cuticle on the sides where the fingers come into contact have rows of metal-enriched and hardened denticles [107], most likely friction-enhancing and grip-improving structures [92,108] which, much like trichobothria, are widely used for taxonomic identification [109].

### Venom delivery system

**Figure 3.**
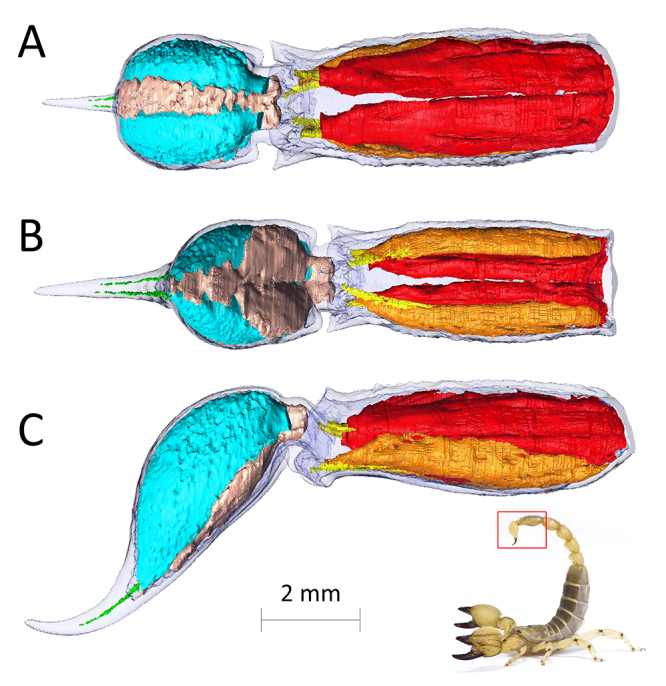
Rendering of the internal anatomy of the V metasomal segment and telson from different points of view. **(A)** Ventral view. **(B)** Dorsal view. **(C)** Lateral view. Note that the telson is dorsoventrally inverted in defensive posture, as in the inset photograph. Cuticle of both telson and V metasomal segment is in transparent blue. Within the V metasomal segment there are a pair of dorsal flexor muscles (*arthrodio-tergal rectus muscles* in orange) and the pair of ventral retractor muscles (*lateral arthrodio-sternal muscles* in red) and the apodemes (yellow) connecting the two antagonistic muscles to the base of the telson. Within the telson are located the paired venom glands (cyan), each ending with its venom duct (green) and separated by a layer of muscles (salmon) responsible for the squeezing of the venom gland against the cuticle and permitting the venom to flow out of the gland lumen. The species used for this µCT scan is *Neochactas delicatus* (Chactidae), but the species shown in the inset is *Scorpio maurus* (Scorpionidae).

The unique metasoma is undoubtedly a scorpion’s most distinctive feature. Evolved for both predatory and defensive purposes [110], this five-segmented structure carries the venomous telson, which consists of the venom vesicle containing two paired venom glands, and the sharp aculeus (Figure 3). 

Metasomal segments develop later than anterior appendages due to the anterior-posterior migration of the growing region [88,89]. In the early stages of development, the metasomal segments are flattened and undifferentiated and the telson is rounded and bilobed. In the later stages of the development, the metasomal segments swell and the telson tapers distally to form the aculeus [89]. 

The joint architecture and muscle organization together give the metasoma a high degree of freedom of movement [111] which is fully achieved after the second instar [89].

The metasoma musculature has been described in [93,94] and partially by [112]. The last segment of the mesosoma contains antagonistic muscles causing protraction and retraction of the metasoma (respectively the *median antero-posterior muscle* and the *arthrodio-tergal rectus muscle*), and two muscles originating dorso-laterally and inserting ventrally at the base to the first metasomal segment (the *arthrodio-tergal obliquus muscles*). These last two antagonistic muscles allow the metasoma to be moved laterally. The metasomal segments I, II and III possess the same muscle distribution: ventrally, a large medial bundle (*median antero-posterior muscle*) and two lateral muscles, (*lateral arthrodio-sternal muscles*). All these muscles originate on the anterior segment and insert on the posterior one. Dorsally, there are four muscles originating on the cuticle of the anterior segment and inserting at the base of the posterior segment. Two large medial bundles (*arthrodio-tergal rectus muscles*) and two small oblique ones are located at the side of each segment (*arthrodio-tergal obliquus muscles*). Segment IV and V each have a different configuration. Metasomal segment IV presents ventrally both the *median antero-posterior muscle* and the two *lateral arthrodio-sternal muscles*. Dorsally, only the two *arthrodio-tergal rectus muscles* are present but differently from the more anterior segments, these muscles are much more elongated and narrower. These muscles have the function to flex and extend the metasoma [112]. The V segment has only a pair of dorsal flexor muscles and a pair of ventral retractor muscles responsible for the movement of the telson (respectively named arthrodio-tergal rectus muscles lateral and arthrodio-sternal muscles ) [52] (Figure 3). The telson consists of a bulbous structure, which contains the two venom glands, and which narrows into the aculeus, the curved and sharp stinger through which venom is injected. 

The cuticle of the stinger and metasoma is thick and covered by granules and several sensory hair-like structures [106,113,114]. In some scorpion species, many cuticular pits containing chemosensory-like setae are present on the ventral and lateral sides of each metasomal segment. This suggests a possible sensory function of the metasoma in these species, similar to the antennae of insects [115]. Soleglad et al. [116] identified minute rows of denticles located laterally on the base of the aculeus in some species, called laterobasal aculear serrations. The function of these is still unknown. Some scorpion species belonging to three different families (Buthidae, Diplocentridae and Vaejovidae) can present a sub-aculear tubercule [117] with important taxonomic value [109] but unknown function [52]. Lourenço [118] suggested that these sub-aculear tubercles may serve as a protection against breakage for particularly long and slender aculei, although simpler reinforcing strategies, such as aculei with a thicker base, already exist within extant scorpions [52]. 

*Venom production and secretion*

Scorpion venom is produced in the secretory epithelium of the two non-communicating venom glands located in the telson. These glands are surrounded by muscle layers radially, while their lateral portions are directly in contact with the endocuticle of the telson (Figure 3). This arrangement is quite conserved in all scorpion families [119-123]. 

Pawlosky [123] divided the scorpion venom glands into primitive and complex, depending on the absence or presence of folds of the secretory epithelium, respectively. Between the muscles and the venom secretory system, a complex matrix of connective tissue is interposed. In the contact area between the lateral telson cuticle and the secretory epithelium a single or multiple layers of cuboid cells are visible. Interior to the connective layer and the cuboid cells lies the basal membrane where the conically shaped secreting cells are located [119-123]. The basal part of these cells contains all the organelles, while the apical part is in touch with the lumen of the venom gland and contains several types of toxin-containing granules. In scorpions, toxin secretion is an apocrine mechanism [119,121,122,124,125], meaning that a portion of the cytoplasm is also secreted into the lumen of the venom gland. Secretory cells seem to be highly specialized in producing one single type of toxin [122]. Each secretory cell contains granules of only one size and type, confirmed by their uniform reaction to laboratory staining techniques [121,126]. Additionally, these different types of granules can be selectively stained. The high diversity of these granules in terms of their reaction to histological stains confirms that different cells produce a different product or mixture of products [122,126]. 

The contraction of the muscles surrounding each venom gland causes the squeezing of the whole gland against the cuticle wall, and the consequent conveying of the venom produced by each gland into a cuticular duct [94]. Both ducts pass through almost the whole length of the aculeus, ending independently before the tip of the aculeus. The ovoid openings, similar to those of hypodermic needles, are found on the dorsal side of the aculeus [53,114,126-129]. Some authors reported that in *Androctonus crassicauda* (Buthidae) [125], *Centruroides sculpturatus* (Buthidae) [119] and *Leiurus quinquestriatus* (Buthidae), the ducts fuse in the terminal part of the aculeus and end in an unique pore.

*Composition and main toxin families*

When extracting venom from scorpions, especially when electrostimulation is avoided, it is possible to observe a transparent to milky-opalescent transition in the venom coming out of the aculeus [45,130-132]. The transparent portion of the venom is generally referred to as pre-venom, while the milky, opalescent portion is considered to be the “true” venom [130]. The pre-venom and true venom present differences in chemical composition, with pre-venom being richer in ions than the true venom, but not in proteins [130,131]. These differences in composition between pre-venom and true venom are likely related to the differences in their appearance. The two fractions also cause different physiological effects when injected, with pre-venom apparently being less toxic than the overall more effective true venom, but still able to induce paralysis and pain [130]. The dichotomy between pre-venom and true venom may be an oversimplification of a continuous or semi-continuous range, with some authors identifying more than two types of venom [131]. 

The protein composition of scorpion venom is particularly complex, with more than 4,500 toxins identified so far across all studied species [133]. Nevertheless, scorpion venoms are not only rich in proteins, but also in nucleotides, amines (serotonin or histamine) and mucopolysaccharides, probably due to the apocrine secretion mechanism of the venom gland cells. However, the role these molecules play in toxicity has yet to be clarified. Previous studies have mainly focused on the peptide components of scorpion venoms. Although a complete overview of the scorpion venom literature is outside the scope of this review, in this paragraph we will highlight some of the best known and important classes of bioactive peptide compounds present in scorpion venoms. These cover about the 75% of the total transcripts obtained from venoms of 37 species belonging to seven different families. The remaining quarter of venom compounds is composed of molecules for which either structural domains and/or function are unknown [134]. For a more comprehensive review of scorpion toxins, refer to [134-138]. 

Four main groups of bioactive compounds are currently known to be present in scorpion venoms: ion-channel binding peptides, enzymes, protease inhibitors and host defense peptides (HDP). Proteomic and transcriptomic analyses performed on venoms and venom glands of scorpions belonging to different families have shown that the most abundant fraction of peptides in scorpion venoms is represented by the superfamily of ion-channel binding toxins. Short-chain ion-channel binding toxins (from 20 to 50 amino acid residues), are specifically active on voltage-gated K^+^ channels. Depending on their length and their folding differences, this class of peptides is further subdivided into several subfamilies. A dedicated database of scorpion toxins active on K^+^ channels (KScTx) is available at [139]. The peptides that bind to voltage-gated Na^+^ channels (NaScTx) have longer chains (50 to 80 amino acid residues). These are classified into α-NaScTx and β-NaScTx, depending on the receptor site they bind to. Calcium channels are targeted by specific scorpion venom peptides known as calcins. These peptides generally compete with the natural ligands of the Ca^2+^ channels affecting muscular contraction [136]. The last members of the family of the ion-channel binding peptides are chlorotoxins (ClTx), altering the conductance of Cl^-^ channels. The toxicological effects of chlorotoxins have been poorly studied so far, but many studies have focused on the potential medical applications of this class of small peptides, especially for the imaging and treatment of aggressive forms of brain cancer generally known as glioma [140-142]. 

Another abundant class of active components of scorpion venoms are the enzymatic toxins. This class of molecules is not as abundant as in other venoms (e.g., snake venom), but its contribution to scorpion venom toxicity is not to be neglected. Phospholipases disrupt the phospholipids in the membranes of cells, causing the lysis of haemocytes and the development of oedemas and release of pro-inflammatory compounds [143]. Another class of enzymes contained in the venom of several scorpion families are the metalloproteases. These proteases are characterized by a bivalent metallic ion (usually the zinc cation) and promote the hydrolysis of proteins present on the cellular membrane. One of the most extensively studied scorpion venom metalloproteases is Antarease. Isolated for the first time from the venom of the Brazilian scorpion *Tityus serrulatus* (Buthidae), this enzyme causes the hydrolysis of the protein regulating the cleavage of the transport vesicles, thus affecting extra-cellular transports, especially in secretory organs like the pancreas [144]. Hyaluronidases represent another class of enzymes widely present in scorpion venom. These enzymes allow a rapid diffusion of the other bioactive compounds through tissues by degrading the extracellular matrix [145]. Even if many of the symptoms generated by scorpion envenomation have a neurotoxic nature, envenomation by species belonging to the medically important genus *Hemiscorpius* (Hemiscorpiidae) represents an important exception. The venom obtained from species belonging to this genus is more similar to snake venom because of the large fraction of enzymes and proteins, and the relatively small fraction of small peptides with affinity for voltage-gated ion channels [146,147]. Venoms produced by *Hemiscorpius* species mainly cause cytotoxic effects, and can cause fatal envenomation while causing little to no pain [148]. 

Protease inhibitors are important components of scorpion venoms that selectively degrade the envenomated organism’s proteases, thus preventing the degradation of the venom peptides injected into the organism’s body, thereby increasing venom efficacy and efficiency [149].

Host Defense Peptides (HDPs) are a large family of small peptides that has been found in all arthropods [150]. They are related to the innate immune defenses of these animals, as most of these peptides have antimicrobial action [151]. This class of proteins is generally divided into two main groups, according to the presence or absence of cysteines in the aminoacidic residual chain [152]. The effect of this relatively abundant class of peptides on scorpion venom toxicity is still not clear, but cases of haemolysis due to HDPs found in the venom of *Pandinus imperator* (Scorpionidae) have been reported [153], possibly suggesting a disruptive effect on blood-clotting mechanisms.

*Scorpion venom evolution*

Venoms evolved independently several times in only a small number of animal taxa [18] where it may be used differently. Venom may have different ecological roles, and the main drivers of its evolution can be different among taxa. In snakes for instance, is widely accepted that venom evolved mainly for predatory purposes [154-156]. Venom may have evolved due to other demands in different groups. Self-defense has been also proposed as driver of venom evolution in fishes and wasps [157,158], intraspecific competition in venomous mammals [81], possibly mating behavior in scorpions [128,159,160] and even for an antimicrobial function in bees [161]. 

The origins of scorpion venom represent an ongoing debate. One of the most widely accepted hypotheses is that toxins may have originated from innate, non-toxic peptides, after a process of gene duplication and neofunctionalization and/or exon shuffling [162-170]. For example, some toxins belonging to the KScTx group present a very high level of structural similarity with defensins and HDPs associated with the innate immune system of arthropods [171-174]. The high similarities between these KScTx toxins and defensins has been used to generate toxigenic compounds able to bind K^+^ channels by modifying a key sequence in defensins [172,173]. The opposite transition, from KScTx to defensin has been observed through experiments of mutagenesis of the genes coding for potassium voltage-gated channel-binding peptides [175]. Interestingly, many toxins with high structural similarity with defensins have been found in other venomous taxa as well (reptiles and mammals), increasing the interest in this class of peptides as an evolutionary ancestor of many different toxins [166,176]. 

*Scorpionism*

“Scorpionism” is the word that is commonly used to refer to fatal envenomation caused by scorpion stings [177]. Annually, around 1.2 million people are stung by scorpions worldwide, and around 3,250 incidents result in fatal envenomation [178]. Incidents are mainly concentrated in tropical countries, where scorpionism is an important but still neglected health issue. The hotspots of scorpionism are in Saharan Africa, the southern and eastern regions of Africa, the Middle East (mainly Iran and Turkey), south India, Mexico, Brazil, and the Amazonian basin area (including the Guianas, Venezuela, and northern Brazil) [178,179]. Ward [180] classified 104 species as potentially harmful (101 Buthidae, 2 Hemiscorpiidae and 1 Scorpionidae), but for only 32 of these fatalities were reported.

## Ecological role of scorpion weapons in feeding, defense and intraspecific agonism

In this section we will review how both scorpion weapon systems are used in three main contexts of usage: feeding, defense and reproduction. 

### Scorpion weapons in feeding

Scorpions are nocturnal generalist predators feeding on a wide spectrum of different prey, consuming mostly arthropods, but also including small mammals and reptiles [128,181-183]. To our knowledge, only two scorpion species are known to have a somewhat specialized diet, apparently preferring spiders as prey items [184,185]. Data about scorpion diet and feeding ecology in the wild is generally sparse [186-191]. Therefore, most of the diet data is based on observations of wild or captive scorpions. Despite this lack of data about diet and feeding ecology, feeding behavior has been studied in almost all scorpion families. Scorpions have very limited vision [128] and prey localization mainly relies on the detection of vibrations and chemical cues. To detect soil-borne vibrations, scorpions rely on slit sensilla, which are mechanoreceptors present on the tarsi of their walking legs [192-196], and the chemo-mechanic receptors on the pectines, which are also used to detect chemical cues [193,197-205]. Scorpions seem not to use trichobothria to locate their prey by vibration (e.g. a walking prey) [206].

Once the prey has been detected, scorpions always use their chelae to grab it. In experiments where both chelae were blocked with wax, scorpions managed to grasp the prey using only the chelicerae [207], showing remarkable plasticity of their predatory behavior repertoire [208]. Once the prey has been grasped, scorpions may or may not use the stinger to inject venom in their prey to subdue it [187,209-213]. Stinger use in scorpion feeding behavior is highly correlated with prey size [200,213-215] and resistance [200,209,212,216], ontogenetic state of the scorpion [211,217] and chela morphology, with species with robust chelae seldom using the stinger to subdue their prey, using only crushing force to incapacitate the prey [211,212,218-221]. 

When a scorpion stings the prey, the telson is projected anteriorly with the metasoma, and the aculeus repeatedly touched to the body of the prey until a soft spot suitable for piercing is found [52,187,210,216,222,223]. Several authors described that after the first sting, scorpions remain motionless for several minutes, most likely waiting for the neurotoxic effects of the injected venom [200,210,223,224]. If the prey keeps struggling, further stinging events can be observed [208,223,225]. Once the prey is successfully incapacitated, scorpions use their chelae to further manipulate it, with several studies showing that scorpions prefer to orient the prey with the head towards their chelicerae before starting to consume it [187,210,219,223,226].

The venom is mainly used for prey incapacitation rather than killing the prey. In many insect prey, the loss of muscle control subsequent to scorpion venom injection is evident [227-229]. Two different types of paralysis induced by the injection of scorpion venom have been described: one is characterized by involuntary contractions of the muscles, while the other is a flaccid paralysis through inhibition of muscle contractions [230]. These neurotoxic effects are mainly provoked by toxins with high affinity to ion-binding voltage-gated channels [135,136,168,231-236]. Within the members of the two main families of NaScTx, for example, we can find specific toxins that are highly toxic only to insects [229,237,238], toxins that have a high affinity to murine sodium voltage-gated channels [233,239,240], and toxins that show similar affinity for both insect and murine ion channels [241,242]. This differential affinity of venom compounds, and the fact that scorpions are both prey and predators, can potentially explain the differences in toxicity that scorpion venom have on different target organisms [243]. The calculation of the median lethal dose (LD_50_), the dose of a venom needed to kill 50% of the test population, is a technique widely used to quantify venom potency [244]. As scorpions are both prey and predators, measuring the LD_50_ on different target organisms is needed to investigate the toxicity of scorpion venom for both defensive and offensive purposes. Zlotkin et al. [230] calculated the LD_50_ of the venom of several species of Buthids on two different target organisms, and found that when venom from the species *Buthus occitanus paris* (Buthidae) was injected into fly larvae, the LD_50_ calculated was the lowest (i.e., highest toxicity), while the same species had the highest LD_50_ in mice. Similar results have been provided by numerous other studies [241,243,245-247], showing that LD_50_ results are only indicative of relative toxicity in the species that was tested, and provide little indication of toxicity in other, even relatively closely related species. Studies on the ecological relevance of scorpion venom should therefore be carried out on the presumed natural target species. 

Venom is considered a fast-changing phenotype [248]. Snake venom, for example, has been seen to change in composition depending on factors like alterations in the animal’s physiological state and diet [249,250]. In recent years, changes in scorpion venom composition and production following diet alterations have been recorded [251,252]. Pucca [251] observed different peaks in venom profiles obtained from scorpions belonging to the same species fed with different types of prey, suggesting rapid adaptation of venom composition to different prey types. Similarly, Tobassum et al. [252] divided scorpions belonging to the same species into groups and fed each group with a different type of prey, observing significant differences in the volume of venom extracted from each group after the same starvation period, suggesting that some prey items are preferable when higher volume of milked venom is required. 

In the species *Centruroides vittatus* (Buthidae), venom toxicity and composition change depending on the ontogenetic state of the animals. Juveniles appear to have less deadly (higher LD_50_) venom than the adults, at least when using crickets as a target species. This may be mediated by a quantitative rather than qualitative change in expression of the different toxins with ontogenetic state [253]. Additionally, scorpions may select different prey according to the amount of venom in their venom glands. Scorpions from which the venom was extracted less than 24 hours before, avoided feeding on larger prey [254]. 

**Figure 4.**
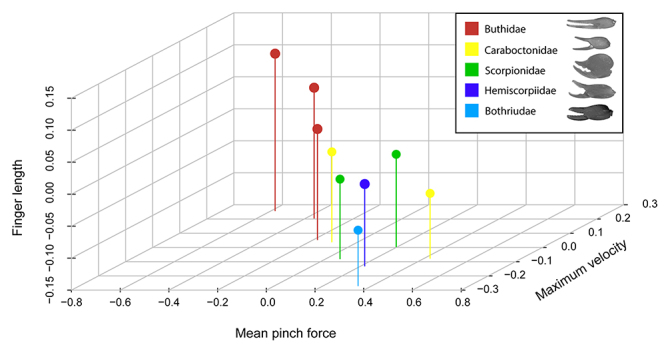
Three-dimensional graph showing the relationships between chela morphology and performance. On the X axis is the pinch force (corrected for overall body size), on the Y axis the size-corrected finger length and on the Z axis the size-corrected maximum closing speed of the chela. A representative member of each family is shown in the legend: *Hottentotta gentili* (Buthidae), *Caraboctonus keyserlingi* (Caraboctonidae), *Pandinoides cavimanus* (Scorpionidae), *Hadogenes paucidens* (Hemiscorpiidae), *Bothriurus chilensis* (Bothriuridae). Species with relatively longer fingers are faster but also weaker than species having shorter fingers. Since the variables have been corrected for overall body size, no units can be given with the axes.

In other pincered taxa, feeding ecology is an important driver for the evolution of the weapons that first touches the food. In decapods (Crustacea) for example, diet seems to be the main factor determining differences in chela morphology and size [96,255-258]. In scorpions, no clear evidence of a similar correlation has been provided yet. However, there may be some rationale to consider diet as a possible driver of scorpion chela evolution. Between different scorpion families, and sometimes even between species belonging to the same genus, chela shape can range from having a stout and robust manus and short fingers to a very slender manus and elongated fingers (Figure 4). Such differences in shape are highly correlated with differences in performance. Pinch force in scorpion species with stouter chelae is much higher than that measured in species having slender chelae [259]. The strongest species also have thicker cuticle [260], probably to withstand the higher stress generated during maximum bite force [261]. Rupturing the exoskeleton of hard-bodied prey requires exerting a significant amount of force, a feat that scorpions with slender chelae may not be able to accomplish without risking breaking their fingers [261]. Lamoral [262] reported Opistophthalmus carinatus (Scorpionidae), a very stout-pincered scorpion, sporadically feeding on terrestrial hard-shelled crustaceans when no other food source is available. Baerg [263] reported that the fine-pincered Centruroides insulanus (Buthidae) feeds on scarab beetles only if these are deprived of the hard elytra. It therefore seems that chela morphology, via performance, may limit feeding on harder prey. 

Whereas in scorpions robust and slender chelae occur in separate species or sexes (see below), in several members of the order Decapoda (Crustacea), a single individual can have one robust (the “crusher”) and one slender chela (the “cutter”) [264-266]. The crusher chela produces a larger pinch force than the cutter, and is mainly used to crack and break the hard shells of the prey, while the cutter chela is mainly used for feeding and prey manipulation [256]. Decapod species feeding on motile, soft-bodied prey, have more elongated and slender chelae [258]. A similar functional specialization may underlie the chela diversity seen in scorpions. Although scorpions do not have different chela morphologies within one individual as some decapods do, the shape of the chelae can differ between sexes and between species. As in decapods feeding on more fleeting and soft-bodied prey, long chelae may aid in prey prehension by allowing a larger gape at the same opening angle, and a higher closing speed of the tips of the fingers, all else being equal. From consideration of lever mechanics, longer fingers (i.e. a longer out-lever) provide a lower mechanical advantage, therefore less force is transmitted from chela muscles to the tip of the movable finger. We recently found a negative correlation between pinch force and chela closing speed [267], which means that species with a stronger grip are also slower (Figure 4). Faster chelae may be a suitable weapon to hunt fast prey, but lever mechanics limits the maximum pinch force, and thus restricts the bearer to soft rather than hard-bodied prey. The negative relationship between chela pinch force and closing speed may thus be a functional trade-off [268] and allows, or may even be driven by, niche partitioning between different species of scorpions.

### Defensive behavior

The chelae and the venom delivery system are not only useful weapons for apprehending and incapacitating prey, but are also very efficient weapons employed in active defense. Experimentally eliciting a defensive response in scorpions can be simple: disturbing a scorpion is usually enough to cause it to show defensive behavior [24,269-271], and the most excitable species just need to feel a puff of air to elicit stinging behavior [45]. A more intense attack may be simulated by touching crucial body parts, such as the prosoma [272-274]. Defensive behavior has been shown to differ between species [24,46,204], perceived threat level [275,276] and sex [271,277,278].

It is important to point out that defensive stinging is very different from predatory stinging [52,269]. Defensive strikes are much faster than predatory ones, and do not include the exploratory touching described above. Depending on metasoma morphology, a defensive strike can have an open or folded trajectory [269]. Species with a muscular or elongated metasoma tend to be faster and have a more open trajectory. Closed trajectories have been observed in species having shorter pedipalps. However, comparative motion shape analysis is in its infancy, and the reasons for these differences in trajectory shape between species are not yet understood. 

Likewise, defensive pinching behavior seems to be different from the predatory grasping of prey. Whereas all scorpions always use their chelae in prey prehension, not all species use their chelae in every defensive case, but sometimes limit their defensive response to stinging only [24]. Warburg [204] recorded scorpions belonging to different families fight each other to observe the different strategies used in intraguild competition and predation. He observed that scorpion species that have strong chelae rarely used their stinger and were less prone to sting, while species with slenderer chelae controlled the opponent with the chelae but were always searching for a suitable spot to sting. This may indicate some functional trade-off or compensation in defensive use. Some scorpion species use their stout chelae as protective shields, placing them in front of the chelicerae to protect the head from frontal attacks [46,279]. Some of these species are burrowers and rock crevice-dwellers, and use their large chelae to prevent unwelcome visitors to access their burrow [46]. 

The use of weapons in defensive behavior is correlated with the perceived threat level and may be different between the two sexes. A more intense threat tends to increase the frequency of defensive stinging [275,276,278,280] and, in some cases, also the volume of venom delivered ([272]. However, the opposite trend has also been recorded [274]. In this study, the authors tested how the scorpion, *Hadrurus arizonensis* (Caraboctonidae), applied its venom defensively during a simulated repeated attack, consisting of 10 consecutive challenges. They found that, surprisingly, the tested specimens invested more venom in the early phases of the threat and that the average volume delivered after 10 consecutive stings was only the 8% of the total yield obtained prior to the beginning of the experiments. Inter-sexual differences have been reported in scorpion defensive behavior. In the sexually dimorphic species *Centruroides vittatus* (Buthidae), females show higher stinging frequency than males when the metasoma was grabbed to elicit a defensive response [277]. However, comparable experiments performed with the similarly sexually dimorphic species *Tityus pusillus* (Buthidae) and *Vaejovis carolinianus* (Vaejovidae) showed no differences in stinging frequency between sexes [276,278]. 

The defensive sting is known to sometimes take place without any venom expenditure (a “dry sting”). Dry stings are reported in different venomous taxa and associated with defensive behavior [281-283]. Even if venom is an efficient weapon in defense, it comes with a high energetic cost and its expenditure has to be carefully metered [284]. Replenishment of the venom glands increases metabolic rate by 21% to 39% for a minimum of 72 hours [285,286]. This may temporarily make the scorpion a less-efficient predator and makes it more vulnerable to potential attackers [254,275,285]. Dry stings, by inflicting pain through mechanical damage, may therefore save the energetically costly resource of venom, while still delivering a painful warning [11].

Pain induction is one of the most common strategies applied by living organisms to deter a predator/attacker. Pain could be useful for defensive purposes but, when considering predation, fast pain induction could represent an evolutionary conflict. While on one hand pain induction could be a good strategy to deter predators from pursuing their attack [287,288], on the other hand pain could enhance prey struggling and make establishing a firm grip on the prey more difficult [51,154,288]. A venom that is used both to incapacitate prey and deter predators should therefore induce pain in its main predators, and paralysis or death in prey. This requires specific toxins for each of these tasks to be present in the venom.

The pain response is mediated by receptors belonging to the nociception system. These sense and transmit environmental stimuli like changes in temperature, mechanical stress or chemicals concentration to the central nervous system, allowing the organism to take action to avoid further damage. The transient receptor potential (TRP) channels are transducers of the nociception system and associated with the pain-inducing response [289-291]. It is not thus surprising that several pain-generating toxins isolated from scorpion venom have a high affinity for TRP channels [134,292-294]. Some specialized scorpion predators like grasshopper mice and bats [295-298] possess an altered molecular configuration of other voltage-gated channels belonging to the nociception system which provide some immunity to the lethal and algogenic effects of scorpion venom [297,299,300]. The pain-inducing effects of scorpion venom are generally evaluated through injection of aliquots of crude venom into the plantar region of mice hind legs [301]. In these tests, pain effects are evaluated by the time the mice spend licking their paws. By using this assay it has been possible to show that buthid scorpion venoms are more painful than venoms of non-buthid scorpions [302], and that, in *Centruroides vittatus* (Buthidae), males are more painful than females [271]. Moreover, even if predators generally prefer to feed on scorpions that inflict less painful stings, significant consumption of more painful species has been reported [300]. When under a strong predatory pressure, however, scorpions can rely on the rapid phenotypic plasticity of their venom to develop an effective defense. When a scorpion is continuously exposed to the presence of a mammalian predator, the production of anti-mammalian toxins in its venom increases [303]. 

Some species of the genus *Parabuthus* (Buthidae) apply their venom externally, as a toxungen rather than a venom [50]. These species are able to spray their venom toward their attacker, similar to the “spitting” behavior of some cobra species [45,46,272,304]. The first symptom following contact of the venom with the human eye is immediate pain [304]. These “venom-spraying” events are unambiguously voluntary, and their use depends on the level of threat perceived by the scorpion. Nisani and Hayes [45] showed that spraying events occurred only if the scorpions were grabbed with tweezers and not when the defensive response was elicited by simply blowing puffs of air on the animals. Moreover, *Parabuthus* species have never been reported to spray venom on their prey during feeding trials [209,213]. Spitting cobras [40,41,154], vinegarroons [44,305], and other animals like bombardier beetles [42] and earwigs [306] likewise spray toxins to deter attackers/predators, but do not use them to incapacitate their prey. 

Another very peculiar defensive use of the metasoma and telson has been observed in some species of the genus *Ananteris* (Buthidae). When grabbed with tweezers, members of this genus are able to cast off their metasoma which also contains their hindgut [307,308]. Similarly to autotomized lizard tails, autotomized metasomas continue to move for a few seconds. Differently from lizard tails, no regeneration has been ever observed. Metasoma autotomy decreases predatory success [309] but it has been observed that acaudate males survived for several months and mated, thus clearly increasing fitness [308]. 

### Mating behavior and sexual dimorphism

While weapons are used for the same purpose by the two sexes in predatory and defensive behavior (be it sometimes to different degrees [277]), in intraspecific competition and mating behavior, members of each sex may use their weapons to accomplish different tasks.

*Male-male antagonism*

Adult scorpions change their behavior during the mating season. Males become more vagrant and actively look for a partner [310-312], which also leads to a higher chance of intrasexual encounters. Literature accurately reporting intrasexual contests between male scorpions is practically non-existent, with the only formal description of one of these events reported for the species *Hadrurus arizonensis* (Caraboctonidae) [78]. In this species, intraspecific contests are divided into three phases: (i) alert phase, (ii) contact phase, and (iii) contest phase. In the alert phase, the opponents face each other with both pedipalps and metasoma raised up. Differently than when performing defensive alert postures, during the intraspecific alert phase both opponents show unique behaviors like metasoma wagging (personal observation YS in *Tityus pachyurus,* Buthidae) and a fast shaking of the whole body without leg movements called “juddering”. These behavioral units have been extensively characterized in the literature on scorpion mating behavior [195,215,313,314] but, due the lack of studies in this topic, are never reported in male-male competition. During the contact phase, scorpions grab each other with their pedipalps. In the contest phase, they try to grab the metasoma of their opponent or, alternatively, try to flip it on one side [78]. During these contests, no actual stings have ever been reported, and the whole behavior seems to be ritualized. The contest ends when one of the competitors holds its position while the other one retreats [78,315].

*Courtship and mating*

When a mature male encounters a female, courtship generally happens. Courtship and mating behavior has been extensively studied in several families of scorpions, and a few taxon-specific differences in the various phases of the courtship ritual have been reported (e.g. the presence/absence of cheliceral massage and sexual sting) [128,314,316,317]. Courtship generally starts with the male approaching the female. The juddering behavior has been observed in different scorpion families in this phase of the mating ritual, and is associated with the production of vibrations that communicate the position of the male to the female, or that help the male control the female’s aggressiveness [195,215,313,314]. The male then attempts to grasp the female’s pedipalps. The initiating phase is very dynamic, and involves males using both their weapons to manipulate the female. The chelae are used to establish a firm grip on the female’s pedipalps to control her movements, which is essential for the next phases of the mating. At the same time, in several species the male rubs, clubs, and even stings the female. After this first contact, the male starts to guide the female by performing specific movements in a dance-like ritual called *promenade à deux*. During this phase, the male moves forward and backwards, dragging the female until he finds a suitable place to deposit the spermatophore. The spermatophore is a stalk-like structure extruded from the male’s genital opening, that serves as a pedestal for the sperm. Once the spermatophore has been deposited, the male guides the female on it, and as soon as the female takes up the sperm package from the spermatophore (leaving only the pedicel anchored to the substrate), the pair separates, ending the mating. This is just a brief illustrative summary of the main phases of scorpion courtship. For more inclusive literature please refer to [159,160,310,311,314,316].

The degree of sexual dimorphism in scorpions is highly variable. Simplifying the classes created by Koch [318] on Australo-Papuan scorpions, and later by Polis [128], it is possible to identify two main patterns of sexual dimorphism in scorpions:

- Differences in body size but not in shape of secondary sexual characters.
- Differences in shape and size of secondary sexual characters.

Sexual size dimorphism in scorpions has been reviewed by McLean et al. [14], with females being generally larger than males probably due to selection on fecundity and to the direct contribution of developing embryos to body size [277]. However, few exceptions of male-biased size dimorphism are present, like in the cases of *Liocheles australasiae* (Scorpiondae) [318] and *Tityus trinitatis* (Buthidae) [316,319]. In the scope of this review, the differences in shape and size of secondary sexual characters deserve particular focus. 

The greatest differences in shape and size of sexually selected characters in scorpions mainly lie in their weapons. Sexual dimorphic species of scorpions generally present differences in chela shape and/or size between the two sexes. In these species, one of the sexes tends to have more robust chelae, while the other sex generally presents a slenderer chela morphology. 

In the members of the families with males having the more robust chelae (e.g., some Buthidae, Scorpionidae), a potential advantage for males may be more space for muscles, and therefore a larger pinch force. This, together with a different distribution and shape of the denticles on the fingers, would provide a firmer grip on the female’s pedipalps during the courtship, or aid to defeat weaker male competitors. A more bulbous chela may also provide less opportunity for male competitors or unwilling females to find purchase. 

In some species the males have more elongated and slender chelae than females [320]. This pattern is most extreme in some members of the families Chactidae [321], Scorpionidae [322] and Buthidae [323,324]. In sexual dimorphic buthid species, the elongation of both pedipalps and metasoma occurs in the last or second-to-last moult [325]. Slenderer chelae are associated with lower bite force in males [321], suggesting that in these cases, selection for higher biteforce is not the main driver determining chela morphology. Studies of the mating ritual of *Centruroides margaritatus* (Buthidae) and *Chactas reticulatus* (Chactidae) [324,326] show that males of these species use their elongated chelae similarly to other scorpions. To date no functional study has shown which of the potential functions and advantages is the driver for sexual dimorphism of chela size in a particular species. Having such elongated appendages may permit to the two sexes of the same species to feed on different prey and exploit a different foraging niche, lowering inter-sexual competition [321], although this cannot be the primary driver of sexual dimorphism.

In some species of Scorpionidae, Hemiscorpidae and Buthidae, males have a prominent tooth on the dorsal side of the movable finger, which is thought to enhance grip on the female’s pedipalps. Once the female has been grasped, her fingers are generally placed in the space between the manus and the tooth, with the latter blocking the retreating of female’s fingers, thus reducing the chances of them slipping out [327]. In Bothriuridae, males present a spine-like apophysis close to a depression present on the surface of their manus. This depression has the function to create a *cul de sac* for the female’s fingers, distally closed by the apophysis [328]. A similar depression in the manus, most likely serving the same function, is also present in adult males of *Pandinoides cavimanus* (Scorpionidae).

The venom delivering system is also a character that may be highly variable in sexually dimorphic species. The general trend is that males have a slenderer, more elongated metasoma, and a more swollen telson than females. Having a longer metasoma has not been related with performance improvements in either locomotion activity or frequency of stinging [277]. However, a longer metasoma allows faster strikes [269] which can be useful in performing quick defensive responses, especially during the mating season, when the increased sexual vagrancy of males increases chances of predator encounters.

A longer metasoma in males may be advantageous during courtship. Males may sting or club the female while keeping her at a greater distance, thus reducing the chances of being stung by an aggressive partner. This behavior most likely has the function of reducing the aggressiveness of a reluctant female [128,159,160,311,314,316,329]. Whether venom injection occurs during sexual stings is still not clear. However, Jiao and Zhu [329] hypothesized that males may deliver a “dry” or a “wet” sting according to the level of aggressiveness of the female. Moreover, differences in telson shape [320,327,330] and venom between sexes have been found in several species of scorpions performing sexual stinging [331-334], with male venoms possessing some unique venom components. In the species *Scorpio maurus* (Scorpionidae) however, females present a more complex venom profile than males [335]. The role of sex-specific toxins in reproductive ecology has not been investigated yet.

 Some authors have hypothesized that differences in the length of the metasoma may be useful in sex recognition in case of intraspecific encounters [316,336]. However, metasoma grabbing in the early stages of mating has also been reported in species where the sexual dimorphism is not very marked [159]. 

In Bothriuridae, males possess accessory glands located on the dorsal side of the telson, with the function of producing secretions when the male rubs the metasoma onto the female’s body [337-340]. According to Peretti [338], these secretions have the function of increasing female’s receptivity. In *Bothriurus bonariensis* (Bothriuridae), the composition of these secretions has been found to change depending on the population analysed [339]. Another example of sexually dimorphism in telson shape is given by the genus *Anuroctonus* (Chactidae). Males of this genus have a secondary bulb of unknown function at the base of the aculeus, which is absent in females [128,341].

## Conclusion

Scorpions use their chelae and venom delivery system in the most fundamental aspects of their ecology. The two weapon systems of scorpions perform in different contexts of usage (predation, self-defense and intrasexual competition), and in some cases interact. How these interactions evolve in different species, or whether there is a trade-off between the weapon systems, is not yet resolved. Despite recent progress in functional studies, several topics still remain underexplored. Of course, a disproportionate fraction of the literature is devoted to venom research. The importance thereof is unambiguous for advances in human health as potential new medicine, as well as in the treatment of scorpion stings as a neglected health risk. Currently basic information on pedipalp anatomy, diet, functional studies of weapons, and intrasexual interactions are sparse or even absent. With the current increased interest in the functional aspects of scorpion weapons, we hope that also these areas will soon reveal new insights in the fascinating ecology of the scorpion weapon constellation. 
